# Radix Paeoniae Rubra stimulates osteoclast differentiation by activation of the NF-κB and mitogen-activated protein kinase pathways

**DOI:** 10.1186/s12906-018-2196-7

**Published:** 2018-04-23

**Authors:** Huey-En Tzeng, Chun-Hao Tsai, Tin-Yun Ho, Chin-Tung Hsieh, Shen-Chieh Chou, Yi-Ju Lee, Gregory J. Tsay, Po-Hao Huang, Yi-Ying Wu

**Affiliations:** 1 Taipei Cancer Center, Taipei Medical University, Taipei, Taiwan; 2 Graduate Institute of Cancer Biology and Drug Discovery, College of Medical Science and Technology, Taipei Medical University, Taipei, Taiwan; 3 Department of Internal Medicine, Division of Hematology/Oncology, Tapei Medical University Shuang-Ho Hospital, Taipei, Taiwan; 4 Department of Orthopedics, School of Medicine, China Medical University Hospital and China Medical University, Taichung, Taiwan; 5 Graduate Institute of Chinese Medicine, China Medical University, Taichung, Taiwan; 6 Department of Pediatrics, Lotung Poh-Ai Hospital, I-Lan, Taiwan; 7 Department of Biological Science and Technology, School of Medicine, China Medical University, Taichung, Taiwan; 8 Institute of Biochemistry, Microbiology and Immunology, Chung Shan Medical University, Taichung, Taiwan; 9 Division of Immunology and Rheumatology, Department of Internal Medicine, China Medical University Hospital, Taichung, Taiwan; 10 Department of Internal Medicine, School of Medicine, China Medical University Hospital and China Medical University, Taichung, Taiwan; 11 Department of Medical Laboratory Science and Biotechnology, China Medical University, No. 91, Hsueh-Shih Rd., Taichung, 404 Taiwan

**Keywords:** Osteoclast, Mitogen-activated protein kinase, Receptor activator of nuclear factor-kappa B

## Abstract

**Background:**

Radix Paeoniae Rubra (RPR), a traditional Chinese herb, has anti-inflammatory and immuno-regulatory properties. This study explored the effects of RPR on stimulation of osteoclast differentiation in RAW264.7 cells and peripheral blood mononuclear cells (PBMC)s.

**Methods:**

The mature osteoclasts were measured by bone resorption assays and TRAP staining. JNK, ERK, p38 and NF-κB inhibitors were used applied in order to verify their contribution in RPR-induced osteoclast differentiation. The NF-κB and MAPK pathways were evaluated by western blotting, RT-PCR and luciferase assay.

**Results:**

RPR induced osteoclast differentiation in a dose-dependent manner and induced the resorption activity of osteoclasts differentiation of RAW264.7 cells and PBMCs. Western blotting showed that RPR treatment induced phosphorylation of JNK, ERK, and p38 in RAW 264.7 cells. Treatment of JNK, ERK, and p38 MAP kinase inhibitors verified the contribution of JNK, ERK and p38. RPR treatment induced c-Fos and NFATc1 protein expression; NF-κB inhibitor treatment and luciferase assay verified the contribution of the NF-κB pathway.

**Conclusions:**

This study demonstrated the interesting effect, in which RPR stimulated osteoclast differentiation in murine RAW264.7 cells and human monocytes.

**Electronic supplementary material:**

The online version of this article (10.1186/s12906-018-2196-7) contains supplementary material, which is available to authorized users.

## Background

Bone remodeling is a regulated, keenly balanced process affected by delicate changes in inhibitory cytokines and proinflammatory factors. These occur predominantly through the TNF-family molecule RANKL (Receptor Activator of NF-kappaB Ligand, a.k.a., OPGL, TRANCE, ODF, and TNFSF11) and its receptor, RANK (TNFRSF11A), which are vital regulators of bone remodeling and essential for the development and stimulation of osteoclasts [1, 2]. Such growth factors and cytokines are associated with the inflammatory processes in rheumatic disease. Various osteopenic disorders, such as Rheumatoid Arthritis, involve amplified osteoclast activity, which can cause the increase in bone resorption, and ultimately crippling damage to bone. Inhibition of RANKL function via Osteoprotegerin (OPG), a natural decoy receptor, can be useful in treating osteoporosis and arthritis. The proposed method establishes an unexpected molecular paradigm that connects bone morphogenesis; variations of these methods create the opportunity to conceive innovative therapies that will impede the bone loss associated with arthritis and osteoporosis [3–6]. Furthermore, apart from the above cytokines (RANKL and M-CSF), recent studies have determined that osteoclastogenesis may also be negatively or positively influenced by many other cytokines or recombinant proteins [6–10].

Radix Paeoniae Rubra (RPR), the dried root of either *Paeonia lactiflora Pall.* or *Paeonia veitchii Lynch*, is a traditional Chinese medicine commonly used for treatment of various diseases in China. RPR has been frequently used to enhance blood circulation, dissipate stasis, and protect the liver [11, 12]. RPR contains triterpenes [13], flavonoids [13], polyphenols [14], and glycoside compounds, such as paeoniflorin, paeonin, benzoylpaeoniflorin, albiflorin, paeonol, and oxypaeoniflorin [15–17], some of which were demonstrated to have some pharmacological effects, including anti-oxidative, anti-atherosclerosis, and anti-inflammatory effects [14, 18].

For thousands of years, RPR has been used to treat various diseases, including hepatitis, diabetes, obesity, traumatic injuries, dementia, and arthritis [19, 20]. The herb contains monoterpene glycosides, galloyl glucoses, and phenolic compounds, and many researchers suggest that it has immuno-regulatory, anti-oxidant, anti-allergic, and anti-inflammatory effects [21–23]. Recently, it has shown that monoterpene glycosides perform a wide variety of biological activities.

## Methods

### Formulation of the aqueous extracts of RPR

RPR (Chishao) was obtained from the GMP pharmaceutical company (Sun Ten Pharmaceutical Co., Taipei, Taiwan). The herb powder (20 g) was extracted with five folds (volume) of distilled water and sterilized by autoclaving (121 °C and 1 atm) for 15 min. Then, the supernatant was collected and further dried under vacuum (76 mmHg, 25 °C). Distilled water dissolved the dried powder (1 mg/ml) before used. The outgoing quality control profiling of Radix Paeoniae Rubra was identified and analysis according to the Taiwan Herbal Pharmacopoeia. The voucher specimen has been deposited in the Institute of Chinese Pharmaceutical Sciences, China Medical University.

### Cell culture

We used the RAW264.7 murine monocytic/macrophagic cell line human peripheral blood mononuclear cells (PBMCs) for the model system of osteoclastogenesis. Cells were maintained as previously described [10]. RAW264.7 cells were purchased from the American Type Culture Collection (ATCC; Rockville, MD, USA). Human PBMCs from healthy donors were separated by gradient centrifugation with Ficoll-Hypaque reagent and were re-suspended in α-MEM supplemented with 10% heat-inactivated FBS.

### Osteoclast differentiation

For osteoclastic differentiation, cells were cultured in the presence of 25 ng/ml murine M-CSF and 50 ng/ml murine RANKL (for RAW264.7, from PeproTech, USA), human M-CSF and human RANKL (for PBMCs, from Peprotech, USA), and RPR. In some experiments, cells were pre-incubated for 40 mins with pharmacologic inhibitor p38/MAPK (SB203580; 10 ng/ml), ERK1/2 (PD98059; 20 μM), and JNK (SP600125; 10 ng/ml) (all from Calbiochem, La Jolla, CA) before RPR was added. In other experiments, cells were pre-incubated for 30 mins with an NFκB inhibitor (NF-κB SN50, cell-permeable inhibitor peptides; Calbiochem, San Diego, CA); caspase-3 specific inhibitor, Z-DEVD-FMK (R&D Systems, Inc., USA); caspase-9 specific inhibitor, Z-LEHD-FMK (BD Biosciences, San Diego, CA); or the general caspase inhibitor, Z-VAD-fmk (Bachem, Bubendorf, Sweden) at a concentration of 20 μM before RPR treatment.

### MTT assay

Cell viability was determined by MTT assay following the procedure described previously [24]. Briefly, cell cultures were treated with varying concentrations of RPR for different set periods. After an incubation with 0.5 mg/ml MTT for 4 h at 37 °C, MTT Formosan was dissolved with the addition of an equivalent cell culture volume of 0.04 N HCl. An ELISA plate reader determined that the absorbance value was 570 nm. The following equation: [OD of solvent-treated cells-OD of compound-treated cells/OD of solvent-treated cells] × 100% was performed to determine cell viability (%).

### TRAP staining

Tartrate-resistant acid phosphatase (TRAP) staining of mature osteoclasts was done as previously described [10]. Briefly, cells were stained with TRAP (Acid Phosphatase Kit 387-A; Sigma-Aldrich, St. Louis, MO) for 30 s, naphthol AS-BI phosphate and tartrate solution for 1 h at 37 °C and then counterstained with a hematoxylin solution. In order to control phosphatase activity that may occur in the background, we opted to use a greater dilution of 1 M tartrate (final 20 mM) and not the tartrate supplied as part of the kit [10]. Intensifying the tartrate dilution suppressed control cells’ staining to allow the RPR and RANKL/M-CSF-treated cells to be positive. The total of TRAP-positive cells and nuclei per TRAP-positive cell in every individual well were respectively counted, followed by the photographing of the morphological features of the osteoclasts.

### Bone resorption assay

The bone resorption assay was conducted as previously described [10]. Cells were seeded into 24-well plates covered by artificial bone slides (BD BioCoat™ Osteologic™ Bone Cell Culture System). After 7 or 14 days of culture(for RAW264.7 or PBMCs, respectively), the wells were washed with DPBS, and the cells within were detached with incubation of 5% sodium hypochlorite for 5 min. The pits that remained in each well were calculated using a microscope and photographed.

### Transfection and luciferase assay

Transfection and luciferase assays were performed based on a previously described method [10]. RAW264.7 cells were seeded into 24-well plates at a density of 7 × 10^4^ cells/well 1 day prior to transfection. Plasmid DNA, lipofectamine reagent and lipofectamine plus reagent were all mixed together in serum free DMEM and moved into the cells according to the recommended procedure of the manufacturer. 20 h after transfection, the cultures were then treated with RANKL (100 ng/ml) or RPR (100 ng/ml) for 12–16 h. The cells were rinsed two times with PBS and lysed in a reporter lysis buffer (Promega, Madison, WI). Following the instructions of the manufacturer, a dual-luciferase reporter assay system (Promega, Madison, WI) then measured the results of luciferase activity.

### Western blot

The Western blot procedure was performed as described previously [10]. Proteins were resolved on SDS-PAGE and transferred to Immobilon polyvinyldifluoride (PVDF) membranes. The blots were blocked with 5% non-fat dry milk in Tris-buffered saline with 0.5% Tween-20 (TBST) for 1 h at room temperature and then probed with p-p38, p-ERK, p-JNK, p38, ERK, JNK (Cell signaling, USA), anti-c-fos, anti-NFATc1 (Santa Cruz Biotechnology, Dallas, TX) for 1 h at room temperature. After three washes, he blots were washed in TBST and developed with horseradish peroxidase conjugated in an anti-mouse antibody (Santa Cruz Biotechnology, Dallas, TX) (diluted in 1:5000) for 1 h in ambient temperature. After another wash, the membrane was subjected to film treated with a chemiluminescence reagent with ECL plus Western blotting reagents (Amersham). Every individual blot was then stripped and re-probed with anti-β actin antibodies to allow the standardization of expression amongst samples. This experiment was replicated three times to corroborate the results of this assay.

### Preparing RNA and real-time RT-PCR

Total RNA and actual RT-PCR were prepared using the SYBR Green incorporation method [10, 25]. A comparative cycle threshold technique using β-actin as the housekeeping gene was used to measure relative gene expression. The primers for NFATc1 were 5′-CGAGCCGTCATTGACTGTGC-3′ (sense) and 5′-GAGCGCTGGGAGCATTCGAT-3′ (anti-sense); 5′- GGTGGAACAGTTATCTCCAG-3′ (sense) and 5′-TGTCTCCGCTTGGAGTGTAT-3′ (anti-sense) for c-Fos; 5′-CGTGCTGACTTCACACCAACAGC-3′ (sense) and 5′-CACTTTTGAAGAGTGCAAACCGCC -3′ (anti-sense) for OSCAR; 5′- CTGTCCTGGCTCAAGAAACAG-3′ (sense) and 5′-CATAGTGGAAGCGCAGATAGC-3′ (anti-sense) for TRAcP; and 5′- GCGGTGGTATTATCTCTTGG-3′ (sense) and 5′-TTCCCTCATTTTGGTCACAAG -3′ (anti-sense) for calcitonin receptors.

### Statistical analysis

Statistical analyses were conducted as previously described [10]. All of the experiments were done in duplicate and the results were averaged. The results were expressed as mean ± SD of averages obtained in at least three experiments. The Student’s *t*-test was used to evaluate variations in the means. A *p* < 0.05 was considered statistically significant.

## Results

### RPR-stimulated osteoclast differentiation from the RAW264.7 cell line and human monocytes

**Fig. 1:**
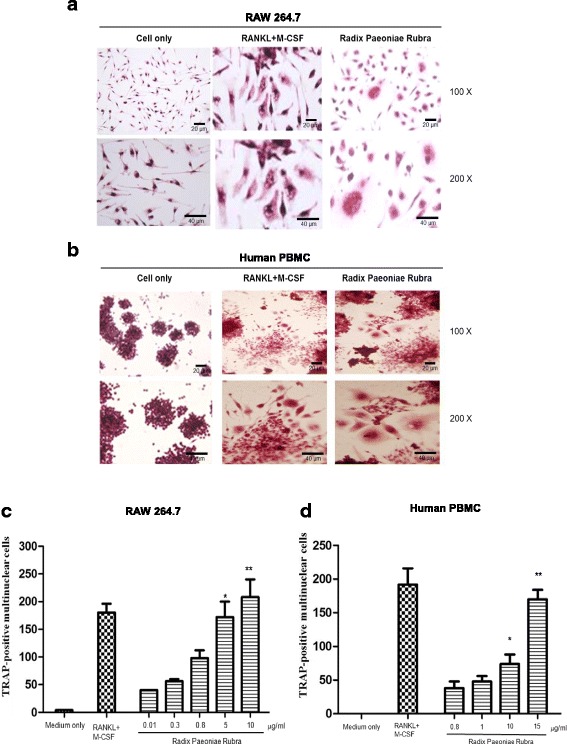
RPR-induced osteoclast-like multi-nucleated cells from RAW264.7 macrophages and human monocytes. **a** RAW264.7 cells and (**b**) human PBMCs were cultured with RANKL+M-CSF or RPR, and then TRAP-stained. **c** RAW264.7 cells and (**d**) human PBMCs were cultured with RANKL+M-CSF or increasing concentrations of RPR, and then TRAP-stained. Data represent the mean ± SD of 3–6 individual experiments. *p < 0.01; **p < 0.001, compared with the control

We first examined the effects of RPR in RAW264.7 cells using TRAP staining. When cultured with M-CSF (25 ng/ml) and RANKL (50 ng/ml), RAW264.7 cells differentiated into osteoclasts, as characterized by TRAP-positive staining. Under RPR treatment, TRAP-positive multi-nuclear cells developed after 7 days of culture (Fig. 1a). Similarly, RPR also stimulated human monocytes (PBMCs) to develop into multi-nuclear TRAP-positive cells within 14 days of culture (Fig. 1b). When treated with different concentrations of RPR, RAW264.7 and human PBMCs differentiated into osteoclasts in a dose-dependent manner (Fig. 1c and d).

### RPR-induced activation of MAP kinases

**Fig. 2:**
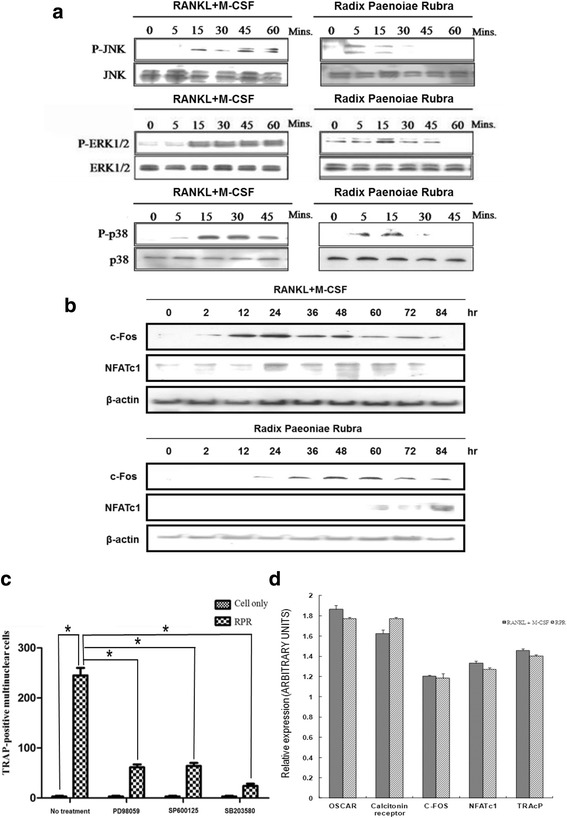
RPR-induced stimulation of MAP kinases. **a** RAW264.7 macrophages were subjected for specified time periods to vehicle RPR (10 μg/ml), or RANKL (50 ng/ml) and M-CSF (25 ng/ml). Cells were then solubilized, and Western blot analysis of p38, JNK, and ERK protein expression was used to examine cell lysates. The top panel of each group shows a trace that denotes the immuno-reactivity of the phosphorylated kinase. The same membrane (shown in the bottom panel) was then exposed and re-probed with the kinase antibody to identify the total kinase protein level. Outcomes represent three separate experiments. **b** Western blotting with Abs specific for β-actin (control), NFATc1, and c-Fos (all from Santa Cruz Biotechnology) was employed to analyze the lysates collected from cultured cells. **c** RPR-induced osteoclast formation required stimulation of JNK, p38, and ERK. Prior to activation with M-CSF and RANKL or RPR (10 μg/ml), RAW264.7 macrophages were pre-treated with vehicle 10 ng/ml SB203580, 10 ng/ml SP600165, or 0.5 ng/ml PD98059 for 20 mins. After culture for 7 days in RAW264.7 macrophages, a TRAP assay was employed on the cells. Data represent the mean ± SD of a minimum of three separate tests; *p < 0.01, RPR treatment versus cell only; #*p* < 0.01, inhibitor treatment versus no treatment. **d** The image shows the consequences of RANKL and RPR on osteoclast gene expression. M-CSF (200 ng/ml) and RANKL (100 ng/ml) or RPR (15 μg/ml) were applied to human monocytes. After culture for 14 days, total RNA was obtained and real-time RT-PCR was conducted for OSCAR, NFATc1, calcitonin receptors, c-fos, and TRAcP. Expression was regulated to that of β-actin; data represent the means ± SD of three repeated wells

The main signaling pathway associated with osteoclast differentiation was investigated. In our previous study, we demonstrated the pivotal roles of MAPKs (JNK, ERK, and p38) in osteoclast development downstream of RANK signaling. In the western blotting assay, we showed that RPR treatment induced phosphorylation of JNK, ERK, and p38 (Fig. 2a). SP600125 (a selective JNK inhibitor), PD98059 (a selective mitogen-activated protein/ERK kinase (MEK) inhibitor), and SB203580 (a selective p38 MAP kinase inhibitor) were applied to verify the contribution of p38 MAP kinase, ERK, and JNK in the behavior of RPR and RANKL. As shown in Fig. 2c, the formation of multi-nuclear cells was constrained by kinase inhibitors, confirming the roles of JNK, ERK, and p38 in osteoclast differentiation induced by RPR.

Since the previous results showed that both the osteoclast-specific transcription factors, c-Fos and NFATc1, were critical for osteoclast differentiation [10], we also examined their expression. RPR treatment induced c-Fos and NFATc1 protein expression in RAW 264.7 cells (Fig. 2b). Real-time RT-PCR was then performed to analyze the levels of c-Fos and NFATc1, and the genes related with osteoclast regulation (calcitonin receptor), fusion (OSCAR), and function (TRAcP; Fig. 2d). The results revealed no substantial variations among cells subjected to RPR and RANKL in downstream osteoclast-specific genes, suggesting a similar activation process.

### Reliance of RPR-induced osteoclast differentiation on NF-κB stimulation

**Fig. 3:**
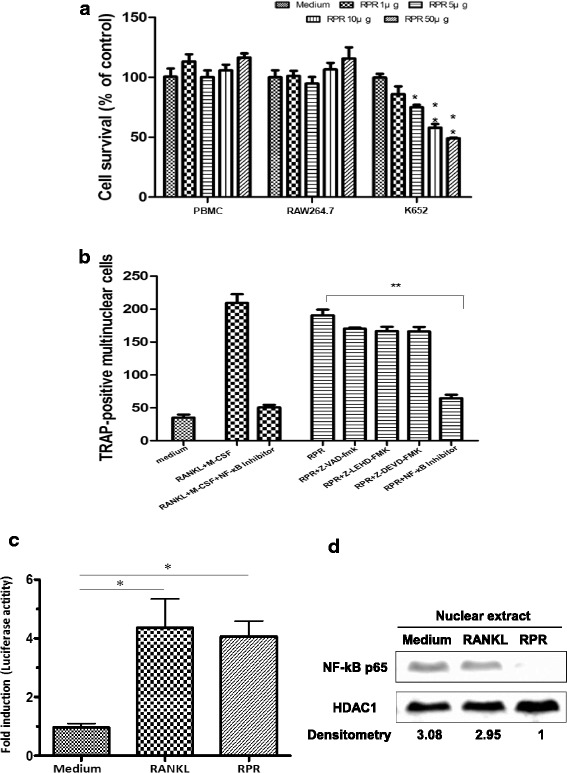
Dependence of RPR-induced osteoclast differentiation on NF-κB stimulation. **a** Human monocytes, RAW264.7 macrophages, and K562 cells were developed with RPR proteins for 18 h. The MTT assay examined cell survival. Data are the mean ± SD of at least three separate tests;*p < 0.01; **p < 0.001, compared with the control for the agent alone. **b** RAW 264.7 cells were given RPR (10 μg/ml) or RANKL and M-CSF, with or without the NF-κB inhibitor (NF-κB SN50), pan-caspase inhibitor (Z-VAD-fmk), caspase-9 specific inhibitor (Z-LEHD-FMK), or caspase-3 specific inhibitor (Z-DEVD-FMK). After incubation for 7 days, a TRAP assay was utilized on the cells. Data are the mean ± SD of at least three independent experiments; **p < 0.001. **c** Murine RAW264.7 macrophages were transfected with an NF-κB luciferase reporter. After 24 h of transfection, cells were treated with RPR (100 ng/ml) or RANKL (100 ng/ml) for 14 h, then lysed for the luciferase assay. A dual-luciferase reporter assay system determined luciferase activity. Data are the mean ± SD of at least three samples. The results reflected at least three independent tests of the same experiment; *p < 0.01. **d** The effects of RPR (100 ng/ml) or RANKL (100 ng/ml) on nuclear NF-κB-p65 levels in murine RAW264.7 macrophages after 30 mins of treatment. RANKL activated NF-κB in RAW264.7. The agent alone did not affect NF-κB stimulation. Each band’s β-actin value normalized the densitometry reading

An MTT colorimetric assay for cell viability was performed to ascertain if RPR induction of osteoclastogenesis impacts osteoclast capability. The results showed that RPR did not exert cytotoxicity on RAW 264.7 or human PBMCs, but displayed signs of cytotoxicity on K562 tumor cells (Fig. 3a) [26].

We investigated whether RPR-induced osteoclast differentiation depends on stimulation of caspase and induction of apoptosis following engagement with RPR. The results indicated that osteoclast precursors opposed RPR-induced apoptosis (Fig. 3b). Furthermore, there were no differences in capacity to stimulate osteoclast differentiation, with or without the caspase-9 specific inhibitor, caspase-3 specific inhibitor, or pan-caspase inhibitor, suggesting that RPR-induced osteoclast differentiation did not depend on the stimulation of caspases.

Conversely, after adding the NF-κB inhibitor to the culture, the capability of RPR to induce osteoclast differentiation was eliminated, implying that RPR-induced osteoclast differentiation activity depends on NF-κB stimulation (Fig. 3b). The NF-κB luciferase reporter plasmid was introduced into RAW264.7, with or without RPR or RANKL, to investigate RPR- and RANKL-induced NF-κB stimulation more closely. RPR or RANKL stimulation induced NF-κB transcriptional activity (Fig. 3c). The medium alone did not affect nuclear NF-κB-p65 levels in RAW264.7 cells. However, addition of RPR or RANKL to the RAW264.7 cell culture media increased NF-κB activation four-fold within 30 mins (Fig. 3d).

### RPR-induced bone resorption activity in RAW264.7 cells and human PBMCs

**Fig. 4:**
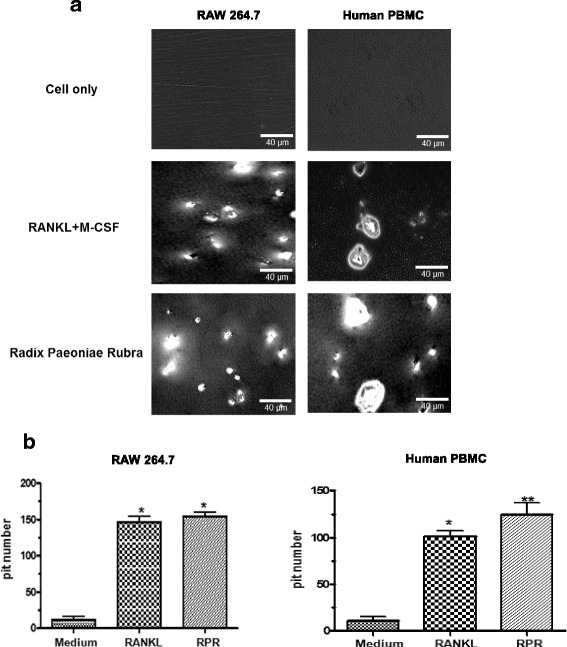
RPR-induced bone resorption activity for osteoclasts. **a** Human PBMCs or murine RAW264.7 macrophages were placed on a synthetic bone cell culture and then treated with RANKL and M-CSF or RPR. After 7 or 14 days of culture, cells were separated. Microscopy was used to determine the number of pits in each well, which were photographed. **b** Data are the mean ± SD from three independent tests; *p < 0.01, **p < 0.001, compared with the medium as the singular control

To determine whether differentiated osteoclast-like multinuclear cells induced by YPH-PA3 have similar characteristics to osteoclasts, functional identification was confirmed in an in vitro culture system with synthetic bone coating. The number of pits dissolved by RAW264.7 cells or PBMCs treated with differentiation agents was compared to those not treated. In contrast to RANKL and M-CSF, osteoclasts that differentiated from RPR dissolved even more pits in the synthetic bone coating (Fig. 4a), suggesting higher bone resorption activity. The following chart presents the number of dissolution holes (Fig. 4b).

## Discussion

RPR (Chishao) was obtained from the GMP pharmaceutical company. The outgoing quality control profiling of Radix Paeoniae Rubra was identified and analysis according to the Taiwan Herbal Pharmacopoeia. One of the preliminary exclusions of animal drugs and toxic drugs. And there are not potential side effects with human in recent research. RPR is used to encourage blood circulation, eliminate blood stasis, reduce fever, cool blood, eliminate stagnant blood, and minimize swelling. Furthermore, RPR also has anti-inflammatory and immune-regulatory usages. The bioactive elements of the plant’s root include a variety of monoterpene glycosides, galloyl glucoses, and phenolic compounds [27, 28]. Furthermore, RPR also has anti-inflammatory and immuno-regulatory usages. RPR-induced K562 tumor cells developed apoptosis because caspase-3 mRNA and caspase-9 mRNA were increased [26]. However, it is unclear if the signaling pathways are an integral part of RPR-induced osteoclast differentiation.

RPR is already known to activate RAW264.7 macrophages and human PBMCs to differentiate into osteoclasts. However, the concentration facilitating optimal differentiation remains unknown. A dose-dependent design (Fig. 1c and d) was used to demonstrate RPR’s novel and unique behavior in osteoclastogenesis, and offer new understanding of the molecular system correlating osteo-immunology with the immune response associated with osteoclasts.

Activation of MAP kinase is a vital step in osteoclastogenesis [29]. In this study, we found that RPR stimulates osteoclast differentiation through a signaling pathway of MAP kinases, which is the same mechanism as TRAIL [30] or ribosome-inactivating protein B-chain [10].

This experiment further employed inhibitors to verify the connection between JNK, ERK, and p38 MAP kinase regarding the behavior of RPR. The differentiation of murine RAW264.7 cells into TRAP-positive multi-nuclear cells was impeded by these kinase inhibitors. The data revealed that NF-κB, p38 MAP kinase, ERK, and JNK signaling pathways are vital to RPR’s osteoclastogenic impact. Similar stimulation of MAP kinases caused the same patterns of expression of vital transcription elements, namely, NFATc1 and c-fos. These can help clarify that RPR function is similar to RANKL in osteoclast differentiation.

The use of an in vitro culture system provides evidence that RPR is an innovative effector molecule that improves the development of osteoclast-like cells. This study also defines the vital function of NF-κB, p38 MAP kinase, ERK, and JNK signaling in inducing osteoclastogenesis, and is the first to test RPR in osteoclast differentiation.

Using MS/MS, we identified the major components from aqueous RPR extract (Additional file 1: Figure S1). gallic acid (1), oxypaeoniflorin (2), albiflorin (3), paeoniflorin (4), benzoic acid (5) and benzoylpaeoniflorin (6) were identified from the aqueous extract of RPR (Additional file 2: Figure S2). However, galloylpaeoniflorin was not found in either paeoniflorin or benzoic acid, based on the retention time under the same solvent conditions [31]. In future studies, we would like to evaluate the osteoclastogenesis properties of the above components.

## Conclusions

This study investigated the biological function of RPR in osteoclast formation. RPR was shown to induce monocyte/macrophage lineage precursor cells to differentiate into osteoclast-like cells in both murine RAW264.7 cells and human PBMCs as RANKL and M-CSF contrasts.

## Additional files

Additional file 1: **Figure S1.** Representative base peak chromatograms of aqueous extract of RPR positive (A) and negative (B) ion modes obtained from Bruker HCT Ultra Ion Trap MS spectrometer. Detailed experimental conditions were shown in the text. (PDF 10 kb)

Additional file 2: **Figure S2.** Chemical structures of the major components identified from the aqueous extract of Radix Paeoniae Rubra. (PDF 7 kb)

## Abbreviations

MAP kinase: Mitogen-Activated Protein Kinase
M-CSF: Macrophage colony-stimulating factor
PBMCs: Peripheral blood mononuclear cell
RANK: Receptor for activation of NF-κB
RANKL: Receptor for activation of NF-κB ligand
RPR: Radix Paeoniae Rubra
TRAP: Tartrate-resistant acid phosphatase
