# Supplementary figures and images for: Radix Paeoniae Rubra stimulates osteoclast differentiation by activation of the NF-κB and mitogen-activated protein kinase pathways
Source: BMC Complement Altern Med. 2018 Apr 23;18:132. doi: 10.1186/s12906-018-2196-7 (PMC5913877; doi:10.1186/s12906-018-2196-7)

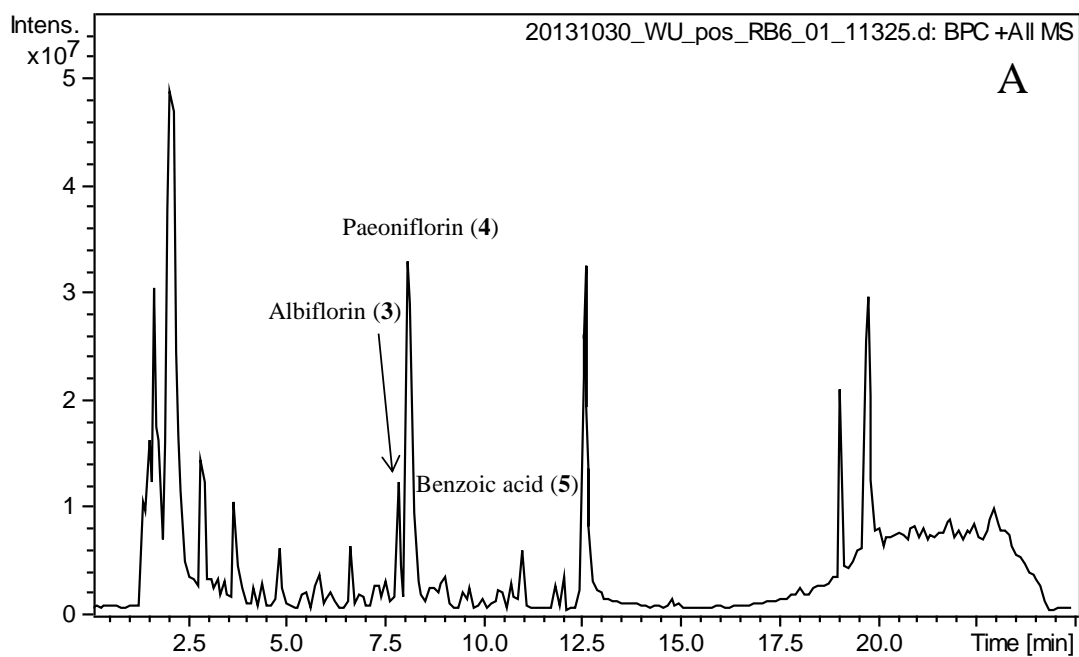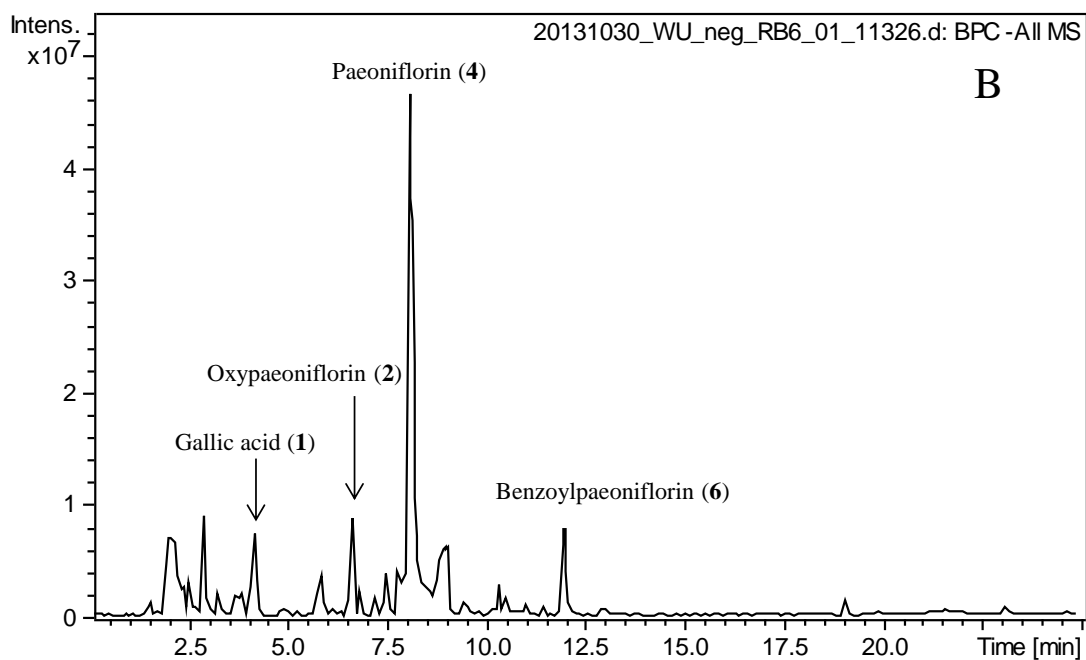

Supplement: Supplementary file 1 — Figure S1. Representative base peak chromatograms of aqueous extract of RPR positive (A) and negative (B) ion modes obtained from Bruker HCT Ultra Ion Trap MS spectrometer. Detailed experimental conditions were shown in the text. (PDF 10 kb) [file 12906_2018_2196_MOESM1_ESM.pdf]
