# Supplementary material for: Radix Paeoniae Rubra stimulates osteoclast differentiation by activation of the NF-κB and mitogen-activated protein kinase pathways
Source: BMC Complement Altern Med. 2018 Apr 23;18:132. doi: 10.1186/s12906-018-2196-7 (PMC5913877; doi:10.1186/s12906-018-2196-7)
Supplement: Supplementary file 2 — Figure S2. Chemical structures of the major components identified from the aqueous extract of Radix Paeoniae Rubra. (PDF 7 kb) [file 12906_2018_2196_MOESM2_ESM.pdf]

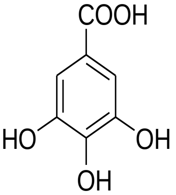

Gallic acid (**1**)

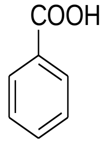

Benzoic acid (**5**)

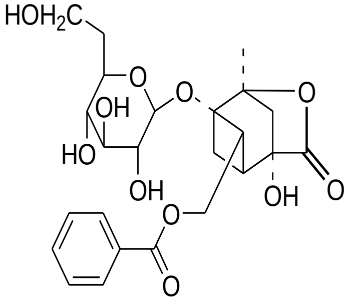

Albiflorin (**3**)

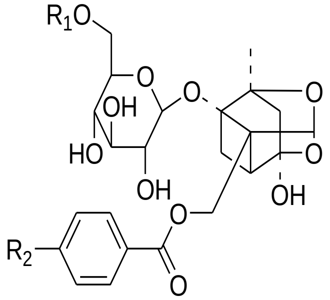

Oxypaeoniflorin (**2**) R_1=H, R_2=OH
Paeoniflorin (**4**) R_1=H, R_2=H
Benzoylpaeoniflorin (**6**) R_1=C_7H_5O, R_2=H
